# Comparison of Vector Competence of *Aedes mediovittatus* and *Aedes aegypti* for Dengue Virus: Implications for Dengue Control in the Caribbean

**DOI:** 10.1371/journal.pntd.0003462

**Published:** 2015-02-06

**Authors:** B. Katherine Poole-Smith, Ryan R. Hemme, Mark Delorey, Gilberto Felix, Andrea L. Gonzalez, Manuel Amador, Elizabeth A. Hunsperger, Roberto Barrera

**Affiliations:** 1 Dengue Branch, Centers for Disease Control and Prevention, San Juan, Puerto Rico; 2 Division of Vector Borne Diseases, Centers for Disease Control and Prevention, Fort Collins, Colorado, United States of America; United States Army Medical Research Institute of Infectious Diseases, UNITED STATES

## Abstract

**Background:**

*Aedes mediovittatus* mosquitoes are found throughout the Greater Antilles in the Caribbean and often share the same larval habitats with *Ae. Aegypti*, the primary vector for dengue virus (DENV). Implementation of vector control measures to control dengue that specifically target *Ae. Aegypti* may not control DENV transmission in Puerto Rico (PR). Even if *Ae. Aegypti* is eliminated or DENV refractory mosquitoes are released, DENV transmission may not cease when other competent mosquito species like *Ae. Mediovittatus* are present. To compare vector competence of *Ae. Mediovittatus* and *Ae. Aegypti* mosquitoes, we studied relative infection and transmission rates for all four DENV serotypes.

**Methods:**

To compare the vector competence of *Ae. Mediovittatus* and *Ae. Aegypti*, mosquitoes were exposed to DENV 1–4 *per os* at viral titers of 5–6 logs plaque-forming unit (pfu) equivalents. At 14 days post infectious bloodmeal, viral RNA was extracted and tested by qRT-PCR to determine infection and transmission rates. Infection and transmission rates were analyzed with a generalized linear model assuming a binomial distribution.

**Results:**

*Ae. Aegypti* had significantly higher DENV-4 infection and transmission rates than *Ae. mediovittatus*.

**Conclusions:**

This study determined that *Ae. Mediovittatus* is a competent DENV vector. Therefore dengue prevention programs in PR and the Caribbean should consider both *Ae. Mediovittatus* and *Ae. Aegypti* mosquitoes in their vector control programs.

## Author Summary

Dengue is a potentially life-threatening tropical disease caused by four serotypes of virus, dengue virus 1, -2, -3, and -4. Worldwide, as many as 390 million people become infected with dengue virus each year after being bitten by infectious *Aedes* mosquitoes. Unfortunately, there is no commercially available vaccine to prevent dengue; so, dengue prevention is attempted by controlling *Aedes* mosquitoes. Since the *Aedes aegypti* mosquito is responsible for most dengue virus infections worldwide, most dengue control efforts target this mosquito. However, *Aedes mediovittatus*, a common mosquito in the Caribbean, may also transmit dengue virus in Puerto Rico. Our goal was to compare dengue virus transmission by *Aedes mediovittatus* and *Aedes aegypti* mosquitoes for four serotypes of dengue virus. In the laboratory, we exposed *Aedes mediovittatus* and *Aedes aegypti* mosquitoes with dengue virus-1–4. We found that similar numbers of *Aedes mediovittatus* and *Aedes aegypti* mosquitoes became infected with dengue virus-1–3, but differed in dengue virus 4 infection rates.

## Introduction

Dengue virus (DENV, Family *Flaviridae*, Genus *Flavivirus*) is most commonly transmitted to humans by the bite of an infected *Aedes aegypti* mosquito. Worldwide, *Ae. aegypti* and *Aedes albopictus* are the main vectors for DENV transmission, however *Ae.albopictus* has not been found in Puerto Rico (PR). The most common container *Aedes* mosquito species in PR are *Ae. aegypti* and the Caribbean treehole mosquito, *Aedes mediovittatus. Ae. mediovittatus* mosquitoes inhabit both natural water-holding containers in cooler, shady forested areas and artificial containers in low density housing and rural areas while *Ae. aegypti* mosquitoes are more abundant in areas of high density urban housing [1–3]. Despite the apparent habitat differences between the two mosquito species, vector control personnel in Cuba reported *Ae. mediovittatus* larvae exploiting the same artificial aquatic habitats normally occupied by *Ae. aegypti* mosquitoes after an intensive *Ae. aegypti* elimination campaign [4]. Since *Ae. mediovittatus* mosquitoes inhabit peridomestic containers and feed on potentially DENV-infected humans, they may be potential secondary DENV vectors in PR and the Caribbean [5].

Dengue epidemics in PR have been documented since 1915 and multi-serotype epidemics have frequently occurred on the island [6–8]. To better understand why DENV was being maintained in rural Puerto Rican communities between epidemics, Gubler *et al*. examined the vector competence of *Ae. mediovittatus* a mosquito frequently found in these communities. Vector competence is measured by the number of mosquitoes which become infected and transmit virus following an infectious bloodmeal [9]. Gubler and colleagues compared DENV-1 and DENV-2 infection and transmission rates and reported that *Ae. mediovittatus* were infected with DENV at a higher rate than *Ae. aegypti* [7]. They concluded that *Ae. mediovittatus* mosquitoes were efficient vectors for DENV and may maintain DENV transmission during inter-epidemic periods.

Collectively, mosquito surveillance reports from Cuba and the vector competence work by Gubler *et al*. suggested that vector control efforts that only target *Ae. aegypti* mosquitoes may not be successful in controlling dengue in PR [4, 7]. The paucity of effective vector control methods available to stop dengue epidemics has prompted development of non-insecticidal mosquito suppression techniques to reduce DENV transmission. New non-insecticidal control techniques, lethal traps, refractory mosquitoes, and lethal genetic modifications, target mosquitoes in an attempt to reduce DENV transmission [10–12]. All of these methods control dengue by reducing or modifying the *Ae. aegypti* populations.

Our goal was to expand our understanding of *Ae. mediovittatus* vector competence for DENV by comparing *Ae. mediovittatus* and *Ae. aegypti* DENV infection and transmission rates for laboratory strains of all four DENV serotypes. To compare DENV competence between mosquito species, we exposed *Ae. mediovittatu*s and *Ae. aegypti* mosquitoes with DENV-(1–4) and determined viral titers and infection and transmission rates. To compare the vector competence by DENV serotype, we analyzed the vector competence results within species by DENV serotype.

## Materials and Methods

### Mosquito Collection and Rearing

Mosquito colonies were established in 2012 using *Ae. aegypti* and *Ae. mediovittatus* mosquito eggs collected in ovitraps from the Patillas municipality in PR. Colonies were supplemented with field-collected eggs every six months to maintain characteristics of wild populations. F_5–6_ eggs were hatched and reared to adults. A taxonomic key of PR mosquitoes (CDC Dengue Branch Entomology) was used to verify species identity. Adult mosquitoes were placed into 1m^3^ cages and allowed to mate freely. Both colonies were maintained at 25–27°C, with 75% relative humidity (RH), and a 12:12 light: dark cycle in separate rooms to prevent cross-contamination. Colonies were offered pig’s blood from a local butcher three times per week.

*Ae. mediovittatus* was conditioned two days prior to *Ae. aegypti* to account for its slower development. Eggs were conditioned for 24 hours by placing egg papers on edge in 200ml of water, allowing water to wick onto the eggs. The egg papers (Anchor Paper, Saint Paul, MN) were then submerged for an additional 24 hour period with 0.05g of ground rabbit food (Amigo Supermarket brand, San Juan, PR) to stimulate hatching. To avoid the effects of larval competition on vector competence, 150 larvae were reared in pans containing 1 liter (L) water [13]. Food was provided daily and proportional to nutritional requirements. Mosquitoes were transferred to cages at the pupal stage. Caged adults were maintained with 10% sucrose solution.

### Virus

Laboratory strains of DENV-1 (Hawaii), DENV-2 (New Guinea C), DENV-3 (H87), and DENV-4 (H241) were grown in 33°C adapted C6/36 (*Ae. albopictus*) cells [14]. To prepare stock virus, a 75-cm^3^ flask of C6/36 cells at 80% confluency was infected at a multiplicity of infection (MOI) of 0.01. Cells were incubated for three days at 33°C in Dulbecco’s Modified Eagle Medium (DMEM) supplemented with 10% heat-inactivated fetal bovine serum (FBS), 1% of 7.5% sodium bicarbonate solution, 1%-non-essential amino acids, 1% MEM vitamins, 1% sodium pyruvate. After three days of growth, supernatant was transferred to 75-cm^3^ flask of C6/36 cells at 80% confluency. Five days after transfer of supernatant, stock virus supernatant was harvested and stored with 5% FBS at -80°C. Virus for all infectious bloodmeals was cultured using an aliquot of stock virus and the three day infection, five day passage method just described.

Plaque forming unit (pfu) equivalents were preferred to genome equivalents in saliva specimens, because pfus were better measure of infectious virions and thus risk of DENV transmission. Therefore we used DENV laboratory strains that reliably produced plaques than recent PR DENV isolates in this experiment [15]. DENV was extracted from cultures using QIAamp viral RNA mini kit (Qiagen, Carlsbad, CA), according to the manufacturer’s instructions and tested via quantitative reverse transcriptase polymerase chain reaction (RT-PCR), as described below, to ensure titers of all four serotypes (DENV1–4) were within 1.0 log_10_ pfu equivalents. Virus titers were 5–6 log_10_ pfu equivalents for all infectious bloodmeals. To prepare infectious bloodmeals, virus supernatant from infected cells was harvested, and mixed: 5 parts virus, 4 parts pig blood, 1 part 10.0 mM adenosine triphosphate disodium salt. Prior to mixing with virus bloodmeal suspension, the pig blood was treated with sodium citrate anticoagulant (108 mM sodium citrate, 16 mM citric acid, 16 mM sodium phosphate, 1.5 mM adenine solution).

### Correlation of Real Time RT-PCR and Plaque Assay

We standardized singleplex real-time RT-PCR assay genome copies to pfu equivalents to generate a standard curve which allowed us to report mosquito specimen viral titers in pfu equivalents [16]. Briefly, virus stocks were serially diluted (10^1–^10^6^) in phosphate buffered saline (PBS) with 30% FBS. Half of each dilution was used for plaque assay, and half was extracted and tested by singleplex real-time RT-PCR [15]. Plaque assay serial dilutions were incubated in twelve-well plates (Corning Costar, Corning, NY) with Vero cell monolayers for one hour. An agarose overlay consisting of 1% agarose in 2X Ye-Lah medium was added to the plate. Ye-Lah medium consists of 2x Earle’s balanced salt solution without phenol red in 1L sterile distilled water supplemented with 0.15g yeast extract, 0.75g lactalbumin hydrolysate, 2% FBS, 2x fungizone and gentamycin. Plates were incubated for four days at 37°C 10% C0_2_ then stained with 3.2% neutral red diluted in PBS. Plaques were counted at five days and every day (up to seven days) until no additional plaques were observed. Final plaque counts were correlated with singleplex real-time RT-PCR results and used to generate a standard curve.

For quality control, iQ5 multicolor Real-Time PCR detection system (BioRad) was calibrated using iCycler iQ calibration kit according to manufacturer’s instructions. Real-time RT-PCR results were considered valid when R ≥0.90.

### Mosquito Exposure to Dengue Virus

Approximately 200 mosquitoes per treatment were sucrose-starved for 36 hours and water-deprived for 12 hours prior to bloodfeed. The bloodmeal mixture described in the virus methods was warmed to 37°C using a Hemotek feeding system (Discovery Workshops, UK) and mosquitoes were allowed to feed for one hour. Aliquots of the bloodmeal were taken before and after feeding and tested by qRT-PCR to ensure there was minimal loss of virus titer. After bloodfeeding, fully engorged mosquitoes were retained and maintained 100/carton for 14 days post-bloodmeal at temperature 25°C and 53–83% RH. Insectary temperature and relative humidity were dependent on ambient conditions. Experimental feed rates were 59% and 64% for *Ae. aegypti* and *Ae. mediovittatus*, respectively. Note: *Ae. mediovittatus* mosquitoes fed poorly on DENV-4 bloodmeal so another batch of *Ae. mediovittatus* mosquitoes were bloodfed DENV-4 a day later than the *Ae. aegypti*. Viral titers for both bloodfeeds were between 1–2 × 10^6^ log_10_ pfu. Those data are reported only for the second batch of *Ae. mediovittatus* mosquitoes. There were no infected mosquitoes from the first batch of *Ae. mediovittatus*. At 14 days, the experimental survival rates were 67% for *Ae. aegypti* and 74% for *Ae. mediovittatus*. A representative sample of 60 mosquitoes from each treatment was used for further analysis.

### Artificial Transmission: Saliva Collection

Fourteen days after infectious bloodmeal, mosquitoes were anesthetized with FlyNap (Carolina Biological Supply Company), transferred to a petri dish, and the proboscis of each mosquito inserted into an individual capillary tube. Capillary tubes were 1.1–1.2 mm diameter, 70 µl capacity Thermo Fisher Scientific, Waltham, MA), scored and broken into 2.5 cm lengths and contained approximately 23.3 µl salivation fluid. Salivation fluid was 10% aqueous sucrose (wt/vol) solution supplemented with 10% FBS. Mosquitoes were allowed to salivate into capillary tubes for 15 minutes, and capillary tubes were transferred to individual 1.5 ml microcentrifuge tubes and centrifuged at 3,000x g for 3 minutes to expel fluid then stored at -80°C until tested.

### Mosquito Processing: Vector Competence

Mosquitoes were tested for the presence of viral RNA 14 days after challenge with an infectious bloodmeal. Mosquito saliva and bodies were stored at -80°C until tested. Individual mosquito bodies were homogenized in 0.5ml BA-1 diluent (1x M199-Hank’s salts, 2mM L-glutamine, 0.05M Tris buffer (pH 7.5), 1% bovine serum albumin (pH 7.0), 100 units penicillin, 0.35 mg sodium biocarbonate, 100µg streptomycin, and 1µg Amphotericin B per ml) with 2 Copperhead Premium BBs at four min, frequency 25/s in Qiagen Tissue Lyser (Qiagen). Saliva samples were centrifuged 3,000 rpm, three min with 0.5ml BA-1. RNA was extracted from 200 µl mosquito body samples using Qiagen M48 robot and MagAttract Mini M48 kit (Qiagen) according to the manufacturer’s instructions. Two hundred microliters of each saliva sample was extracted by hand, to minimize loss of low volume saliva samples, using QIAamp viral RNA mini kit (Qiagen), according to the manufacturer’s instructions. All samples were analyzed by singleplex real time RT-PCR standardized to a curve consisting of serial RNA dilutions.

### Data Analysis

All statistical analyses of infection, transmission rates (number of mosquitoes that transmitted DENV/ total number of mosquitoes exposed to DENV), and transmission efficiency (number of positive transmissions/number of mosquitoes infected with DENV) were performed with R software v2.15.1. Comparisons of infection, transmission rates, and transmission efficiency were analyzed using generalized linear models (glm) assuming binomial distribution and logit link. Serotype, species, and their interaction were included as explanatory variables. Multiple comparisons on all pair-wise means using Sidak’s method with simultaneous 95% confidence intervals were conducted on statistically significant effects. In order to determine if higher infection titers were associated with increased likelihood of transmission, transmission status was regressed on body titer and its interaction with species and serotype using a glm assuming a binomial distribution with logit link. Finally, body titers by species and serotype were compared using a glm assuming a Poisson distribution with log link. Graphs representing viral titers were produced with GraphPad Prism 5 and mean, 25^th^ and 75^th^ percentile titers are indicated.

## Results

### Mosquito Susceptibility to DENV Infection

**Figure 1:**
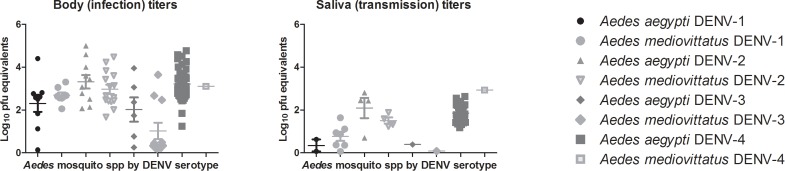
Body (infection) and saliva (transmission) titers of dengue virus-infected *Aedes mediovittatus* and *Aedes aegypti* mosquitoes 14 days after oral challenge with dengue-1 (DENV-1) (HAW), DENV-2 (New Guinea C), DENV-3 (H87), DENV-4 (H241). Non-zero mean titer, 25 percentile titer, and 75 percentile titers are indicated on the graphs.

Twenty-five percent of *Ae. mediovittatus* mosquitoes exposed to DENV-2 became infected with DENV-2 with an average viral titer of 3.0±0.8 log _10_pfu/mosquito at 14 days post infection (dpi) compared to 18% infected with DENV-3 (mean = 1.0±1.3 log_10_ pfu/mosquito), 13% with DENV-1 (mean = 2.7±0.4 log_10_ pfu/mosquito), and 2% with DENV-4 (mean = 3.1±0.0 log_10_ pfu/mosquito) (Table 1, S1–S5 Tables in S1 Text). There were no significant differences in *Ae. mediovittatus* infection rates or viral titers between DENV serotypes. Sixty-two percent of *Ae. aegypti* mosquitoes exposed to DENV-4 became infected by 14 dpi with an average viral titer of 3.2±0.8 log_10_ pfu/mosquito compared to 17% infected with DENV-2 (mean = 3.3±1.0 log_10_ pfu/mosquito), 15% with DENV-1 (mean = 2.3±1.2 log_10_ pfu/mosquito), and 10% with DENV-3 (mean = 2.0±1.4 log_10_ pfu/mosquito) (Table 1, S1–S5 Tables in S1 Text). There was a significant difference in the *Ae. aegypti* infection rate with DENV-4 compared to the other serotypes (p < 0.05) (Table 1). The infection rate for DENV-4 at 14 dpi was significantly higher for *Ae. aegypti* than *Ae. mediovittatus* mosquito species (glm, p < 0.001. Additionally, individual viral infection titers were variable for both species (Fig. 1) thus there were no significant differences between viral infection titers between species for DENV1–3 (Table 1, Fig. 1).

**Table 1:** Infection rates and body titers of *Aedes mediovittatus* and *Aedes aegypti* mosquitoes 14 days post bloodmeal.

**DENV serotype (strain)**	**Mosquito species**	**number mosquitoes infected/total mosquitoes (%)**	**non-zero mean titer ± SD log_10_ pfu/mosquito**
**DENV-1 (HAW)**	*Aedes mediovittatus*	8/60 (13)	2.7 ± 0.4
	*Aedes aegypti*	9/60 (15)	2.3 ± 1.2
**DENV-2 (NGC)**	*Aedes mediovittatus*	15/60 (25)	3.0 ± 0.8
	*Aedes aegypti*	10/60 (17)	3.3 ± 1.0
**DENV-3 (H87)**	*Aedes mediovittatus*	11/60 (18)	1.0 ± 1.3
	*Aedes aegypti*	6/60 (10)	2.0 ± 1.4
**DENV-4 (H241)**	*Aedes mediovittatus*	1/60 (2)	3.1 ± 0.0
	*Aedes aegypti*	37/60 (62)	3.2 ± 0.8

Mosquitoes were exposed to dengue-1 (DENV-1) (HAW), DENV-2 (New Guinea C), DENV-3 (H87), DENV-4 (H241) *per os*.

### DENV Transmission Rates and Viral Titers

Twelve percent of *Ae. mediovittatus* mosquitoes artificially transmitted DENV-1 at 14 dpi with an average titer of 0.8±0.5 log _10_pfu/mosquito compared to 7% transmitting DENV-2 (mean = 1.5±0.3 log_10_ pfu/mosquito), 2% transmitted DENV-3 (mean = 0.1±0.0 log_10_ pfu/mosquito), and 2% transmitted DENV-4 (mean = 2.9±0.0 log_10_ pfu/mosquito) (Table 2, S1–S5 Tables in S1 Text). There were no significant differences in *Ae. mediovittatus* transmission rates between serotypes. Forty-two percent of *Ae. aegypti* mosquitoes artificially transmitted DENV-4 on 14 dpi with an average titer of 1.9±0.4 log_10_ pfu/mosquito compared to 5% transmitted DENV-2 (mean = 2.1±0.9 log_10_ pfu/mosquito), 3% transmitted DENV-1 (mean = 0.3±0.4 log_10_ pfu/mosquito), and 2% transmitted DENV-3 (mean = 0.4±0.0 log_10_ pfu/mosquito) (Table 2, S1–S5 Tables in S1 Text). Significantly more *Ae. aegypti* transmitted DENV-4 (42%) than DENV-1, -2, and-3 (3%, 5%, and 2% respectively) (p < 0.05); there were no significant differences in *Ae. aegypti* saliva titers by DENV serotype. When challenged with DENV-4, significantly more *Ae. aegypti* (42%) were transmitting DENV-4 than *Ae. mediovittatus* (2%) (p < 0.05) (Table 2). There were no significant differences in transmission rates between species for DENV-1, 2, 3 (Table 2, Fig. 1).

**Table 2:** Transmission rates and saliva titers of *Aedes mediovittatus* and *Aedes aegypti* mosquitoes 14 days post bloodmeal.

**DENV serotype (strain)**	**Mosquito species**	**number mosquitoes transmitting/total mosquitoes (%)**	**non-zero mean titer ± SD log_10_ pfu/mosquito**
**DENV-1 (HAW)**	*Aedes mediovittatus*	7/60 (12)	0.8 ± 0.5
	*Aedes aegypti*	2/60 (3)	0.3 ± 0.4
**DENV-2 (NGC)**	*Aedes mediovittatus*	4/60 (7)	1.5 ± 0.3
	*Aedes aegypti*	3/60 (5)	2.1 ± 0.9
**DENV-3 (H87)**	*Aedes mediovittatus*	1/60 (2)	0.1 ± 0.0
	*Aedes aegypti*	1/60 (2)	0.4 ± 0.0
**DENV-4 (H241)**	*Aedes mediovittatus*	1/60 (2)	2.9 ± 0.0
	*Aedes aegypti*	25/60 (42)	1.9 ± 0.4

Mosquitoes were exposed to dengue-1 (DENV-1) (HAW), DENV-2 (New Guinea C), DENV-3 (H87), DENV-4 (H241) *per os*.

### Transmission Efficiency: Transmission Rates for Infected Mosquitoes

Eighty-eight percent (7/8) of DENV-1 infected *Ae. mediovittatus* mosquitoes artificially transmitted DENV compared to 27% (4/15) for DENV-2, 9% (1/11) for DENV-3, and only 1 *Ae. mediovittatus* mosquito transmitted DENV-4 for 100% (1/1) of infected mosquitoes transmitting(Table 3). Sixty-eight percent (25/37) of DENV-4 infected *Ae. aegypti* mosquitoes artificially transmitted DENV compared to 30% (3/10) for DENV-2, 22% (2/9) for DENV-1, and 17% (1/6) for DENV-3 (Table 3). Transmission efficiency between mosquito species was not statistically significant (S3 Table in S1 Text).

**Table 3:** Transmission efficiency of dengue virus infected *Aedes mediovittatus* and *Aedes aegypti* mosquitoes at 14 days.

**DENV serotype (strain)**	**number *Aedes* transmissions / number *Aedes* infections**	***Aedes* transmission efficiency %**	***Ae. mediovittatus* number transmissions/number infections**	***Ae. aegypti* number transmissions/number infections**
**DENV-1 (HAW)**	9/17*	53%	7/8	2/9
**DENV-2 (NGC)**	7/25^#^	28%	4/15	3/10
**DENV-3 (H87)**	2/17*, ^#^	12%	1/11	1/6
**DENV-4 (H241)**	26/38^#^	68%	1/1	25/37

DENV artificial transmission for mosquitoes infected with dengue-1 (DENV-1) (HAW), DENV-2 (New Guinea C), DENV-3 (H87), DENV-4 (H241) *per os*.

* Statistically significant differences for comparisons between serotypes DENV-1 >DENV-3 using Sidak’s method for multiple comparisons with simultaneous 95% confidence intervals.

^#^ Statistically significant differences for comparisons between serotypes DENV-4 > DENV-2 andDENV-3 using Sidak’s method for multiple comparisons with simultaneous 95% confidence intervals.

Transmission efficiency between DENV serotypes was analyzed, independent of mosquito species by pooling data across mosquito species. DENV-1 and DENV-4 are the most efficiently transmitted DENV serotypes. Fifty-three percent of *Aedes* mosquitoes infected with DENV-1 transmitted compared to 12% of mosquitoes infected with DENV-3 (p < 0.05). Sixty-eight percent of *Aedes* mosquitoes infected with DENV-4 transmitted compared to 28% infected with DENV-2 or 12% infected with DENV-3 (p < 0.05) (Table 3). Our analysis indicated that one log_10_ increase in the infection titer of a mosquito did not increase the likelihood that a mosquito could transmit DENV.

## Discussion

This study concluded that *Ae. mediovittatus* mosquitoe*s* are equally competent vectors for DENV-1, DENV-2, and DENV-3 when compared to *Ae. aegypti* [7]. However, *Ae. aegypti* mosquitoes were more susceptible to DENV-4 infection than *Ae. mediovittatus*. This is the first study that compared the competence of *Ae. mediovittatus* and *Ae. aegypti* mosquitoes for all four DENV serotypes. A previous study by Gubler *et al*. also compared both species, however due to differences in methodology including: a higher incubation temperature (30°C) and a different titration method (mosquito infectious dose 50), we cannot directly compare the results between the two studies. Since these are the only studies that have compared locally collected *Ae. mediovittatus* and *Ae. aegypti* mosquitoes, we compared the overall conclusions between the two studies; Gubler concluded that *Ae. mediovittatus* was more susceptible to infection with DENV-1 and DENV-2 than *Ae. aegypti* [7]. In contrast, the infection rates in our study were not significantly different between mosquito species, except for DENV-4.

There are few studies which address the question of vector competence between all four DENV serotypes and these have reported no differences between serotypes [17, 18]. More commonly, vector competence studies compare fewer DENV serotypes and one of these studies reported significantly higher DENV-2 infection and transmission rates than DENV-4 rates in *Ae. aegypti* collected from four different geographic locations [19]. Interestingly, in our study *Ae.aegypti* had the highest competence with DENV-4 compared to any other DENV serotypes. Differences between the Knox study and our study could be explained by frequently reported geographic differences in *Ae. aegypti* competence for DENV [17, 20–24]. When comparing our vector competence results for *Ae. aegypti* to other studies, we noted that *Ae. aegypti* vector competence rates in PR were lower than from other dengue endemic regions [17, 21, 23]. Observed differences in vector competence could be explained by differences in genetic structure of the mosquito population, wildtype virus strains, and variations in experimental methodologies. While these were beyond the scope of our study, we can compare our experimental methodology with other studies. In this study the temperature in the insectary was 3–5°C degrees lower than other comparable studies (25°C). Our insectary was dependent on ambient temperature, and our experiments were performed during low temperature conditions potentially resulting in lower transmission and infection rates. The average temperature during our experiment was 25°C, equivalent to March temperatures in San Juan, PR when typically there are lower number of reported dengue cases [25, 26]. Low temperatures (≤25°C) have been widely reported to decrease infection and transmission rates for DENV by increasing extrinsic incubation period (EIP) [27–30]. Nevertheless, *Ae. mediovittatus* mosquitoes were infected and transmitted a 4 DENV serotypes under low temperature conditions (≤25°C) at rates less than or equal to those observed for the primary vector *Ae. aegypti*.

When we compared transmission rates across DENV serotypes, we observed significantly higher DENV-4 transmission by *Ae.aegypti* mosquitoes. DENV-4 was first introduced to PR from Dominica in 1981 and subsequently caused a large dengue epidemic [31, 32]. Phylogenetic analyses of DENV-4 in the Caribbean reported various selection pressures which could lead to ideal conditions for the transmission of DENV-4 throughout the Caribbean. These conditions included positive selection on the NS2a component of DENV-4 replication complex, maintenance of DENV-4 in the Caribbean region during DENV-4 extinctions in PR, and higher rates of DENV-4 dispersal [33–35]. Carrington *et al*. even suggested that selection pressures on Caribbean DENV-4 isolates could lead to enhanced DENV-4 growth rates in mosquitoes. Furthermore, DENV-4 epidemics have been recently reported in PR and in the U.S. Virgin Islands [36, 37]. Overall recent conditions in the Caribbean have favored DENV-4 transmission, potentially due to mosquito transmission efficiency as determined in our study, positive selection pressure on DENV-4, or low immunity in the human population.

*Ae. aegypti* and *Ae. mediovittatus* have equivalent competence for DENV-1, -2, and-3; however our understanding of the relative contribution of *Ae mediovittatus* to DENV transmission is still incomplete. The difference in transmission rates between DENV-4 exposed versus infected *Ae. mediovittatus* mosquitoes requires further evaluation to determine whether there is a midgut infection barrier. There are other factors including human biting rate, mosquito population density, and EIP, (i.e. vectorial capacity) which were not examined in this study [38]. Both mosquito species can live in close proximity to humans and feed on human blood, however *Ae. mediovittatus* feeds on a broader range of hosts [2, 5]. Analogous to *Ae. albopictus* in global DENV transmission, the broad feeding behavior of *Ae. mediovittatus* mosquitoes may limit their vectorial capacity [39]. Furthermore, our study did not account for vertical transmission (VT) in *Ae. mediovittatus*. Theoretically, mosquitoes infected by VT are competent to transmit DENV 7–10 days earlier than orally infected mosquitoes. Adams and Boots estimated that VT rates must be as high as 20–30% to impact DENV transmission [40]. Previous studies found that *Ae. mediovittatus* VT rates were as high as 20.3% which is within range to hypothetically impact DENV transmission [40, 41]. To determine the role of *Ae. mediovittatus* mosquitoes in DENV transmission in PR, future research should focus on vectorial capacity.

### Conclusions

Dengue is a vector bone disease of major public health importance, so many researchers are developing non-insecticidal mosquito control techniques to reduce dengue transmission. Most of these non-insecticidal control techniques, lethal traps, refractory mosquitoes, and lethal genetic modifications, target primarily *Ae. aegypti* mosquitoes. Eliminating only the primary DENV vector, *Ae. aegypti*, may have unexpected consequences in the presence of other secondary vectors (e.g., *Ae. albopictus* and *Ae. mediovittatus*) that are capable of transmitting DENV. We determined that *Ae. aegypti* and *Ae. mediovittatus* mosquitoes are comparatively competent to transmit DENV1–3 but differ in competence for DENV-4. Our results have several implications for DENV transmission in PR, most interesting are the implications for non-insecticidal control techniques. Since both *Ae. aegypti* and *Ae. mediovittatus* are competent DENV vectors, non-insecticidal mosquito control techniques that target *Ae. aegypti* may not be effective in PR because they do not account for local secondary vectors. If such methods are used to eradicate *Ae. aegypti*, secondary DENV vectors such as *Ae. mediovittatus*, could expand their populations and drive DENV transmission negating the utility of non-insecticidal mosquito control techniques in the elimination of dengue [4]. Consequently, the use of non-insecticidal control techniques to control dengue requires careful assessment of local DENV vectors.

## Supporting Information

S1 Text Correlation of body (infection) titers and saliva (transmission) titers of *Aedes mediovittatus* and *Aedes aegypti* mosquitoes 14 days after oral challenge with dengue-1 (DENV-1) HAW, DENV-2 (New Guinea C), DENV-3 (H87), and DENV-4 (H241). Threshold cycle (Ct) is the RT-PCR cycle at which the signal crossed the baseline amplification threshold.
(DOCX)
