# Supplementary material for: Comparison of Vector Competence of Aedes mediovittatus and Aedes aegypti for Dengue Virus: Implications for Dengue Control in the Caribbean
Source: PLoS Negl Trop Dis. 2015 Feb 6;9(2):e0003462. doi: 10.1371/journal.pntd.0003462 (PMC4319915; doi:10.1371/journal.pntd.0003462)
Supplement: S1 Text — Threshold cycle (Ct) is the RT-PCR cycle at which the signal crossed the baseline amplification threshold. (DOCX) [file pntd.0003462.s001.docx]

**Table S1**

| **Sample data** | | **Body (infection) data** | | | **Saliva (transmission) data** | | |
| --- | --- | --- | --- | --- | --- | --- | --- |
|  |  | **only positive mosquitoes** | | |  |  |  |
| **mosquito** | **DENV** | **Ct** | **titer** | **log** | **Ct** | **titer** | **log** |
| **species** | **serotype** |  | **log10 pfu/** | **titer** |  | **log10 pfu/** | **titer** |
|  |  |  | **mosquito** |  |  | **mosquito** |  |
| *Ae aegypti* | 1 | 26.3 | 5.8E+02 | 2.8 | 38.5 | 2.3E-01 | -0.6 |
| *Ae aegypti* | 1 | 27.0 | 3.8E+02 | 2.6 | N/A | 0.0E+00 | 0.0 |
| *Ae aegypti* | 1 | 36.9 | 1.4E+00 | 0.1 | N/A | 0.0E+00 | 0.0 |
| *Ae aegypti* | 1 | 26.9 | 4.1E+02 | 2.6 | N/A | 0.0E+00 | 0.0 |
| *Ae aegypti* | 1 | 30.1 | 6.8E+01 | 1.8 | 39.0 | 1.7E-01 | -0.8 |
| *Ae aegypti* | 1 | 32.9 | 1.3E+01 | 1.1 | 37.9 | 3.2E-01 | -0.5 |
| *Ae aegypti* | 1 | 27.4 | 3.2E+02 | 2.5 | 33.2 | 4.2E+00 | 0.6 |
| *Ae aegypti* | 1 | 19.7 | 2.5E+04 | 4.4 | 37.9 | 3.2E-01 | -0.5 |
| *Ae aegypti* | 1 | 26.1 | 6.5E+02 | 2.8 | 35.5 | 1.2E+00 | 0.1 |
| *Ae mediovittatus* | 1 | 27.2 | 3.7E+02 | 2.6 | 32.1 | 7.5E+00 | 0.9 |
| *Ae mediovittatus* | 1 | 24.1 | 2.0E+03 | 3.3 | 28.9 | 4.4E+01 | 1.6 |
| *Ae mediovittatus* | 1 | 26.7 | 4.8E+02 | 2.7 | 31.1 | 1.3E+01 | 1.1 |
| *Ae mediovittatus* | 1 | 26.9 | 4.4E+02 | 2.6 | 34.2 | 2.4E+00 | 0.4 |
| *Ae mediovittatus* | 1 | 27.2 | 3.6E+02 | 2.6 | 35.5 | 1.2E+00 | 0.1 |
| *Ae mediovittatus* | 1 | 29.3 | 1.1E+02 | 2.1 | 38.2 | 2.7E-01 | -0.6 |
| *Ae mediovittatus* | 1 | 25.2 | 1.1E+03 | 3.1 | 31.9 | 8.7E+00 | 0.9 |
| *Ae mediovittatus* | 1 | 26.9 | 4.3E+02 | 2.6 | 34.3 | 2.3E+00 | 0.4 |

**Table S2**

| **Sample data** | | **Body (infection) data** | | | **Saliva (transmission) data** | | |
| --- | --- | --- | --- | --- | --- | --- | --- |
|  |  | **only positive mosquitoes** | | |  |  |  |
| **mosquito** | **DENV** | **Ct** | **titer** | **log** | **Ct** | **titer** | **log** |
| **species** | **serotype** |  | **log10 pfu/** | **titer** |  | **log10 pfu/** | **titer** |
|  |  |  | **mosquito** |  |  | **mosquito** |  |
| *Ae aegypti* | 2 | 31.7 | 1.2E+03 | 3.1 | 39.3 | 5.0E+00 | 0.7 |
| *Ae aegypti* | 2 | 33.1 | 6.0E+02 | 2.8 | N/A | 0.0E+00 | 0.0 |
| *Ae aegypti* | 2 | 22.5 | 1.0E+05 | 5.0 | N/A | 0.0E+00 | 0.0 |
| *Ae aegypti* | 2 | 29.2 | 3.9E+03 | 3.6 | N/A | 0.0E+00 | 0.0 |
| *Ae aegypti* | 2 | 36.5 | 1.2E+02 | 2.1 | N/A | 0.0E+00 | 0.0 |
| *Ae aegypti* | 2 | 34.4 | 3.2E+02 | 2.5 | N/A | 0.0E+00 | 0.0 |
| *Ae aegypti* | 2 | 24.4 | 3.9E+04 | 4.6 | 31.3 | 2.8E+02 | 2.4 |
| *Ae aegypti* | 2 | 27.3 | 1.0E+04 | 4.0 | 31.4 | 2.7E+02 | 2.4 |
| *Ae aegypti* | 2 | 30.1 | 2.6E+03 | 3.4 | 29.7 | 6.5E+02 | 2.8 |
| *Ae aegypti* | 2 | 36.2 | 1.4E+02 | 2.1 | N/A | 0.0E+00 | 0.0 |
| *Ae mediovittatus* | 2 | 28.3 | 2.7E+03 | 3.4 | 40.1 | 3.4E+00 | 0.5 |
| *Ae mediovittatus* | 2 | 31.9 | 3.8E+02 | 2.6 | N/A | 0.0E+00 | 0.0 |
| *Ae mediovittatus* | 2 | 24.0 | 3.0E+04 | 4.5 | N/A | 0.0E+00 | 0.0 |
| *Ae mediovittatus* | 2 | 27.6 | 4.1E+03 | 3.6 | N/A | 0.0E+00 | 0.0 |
| *Ae mediovittatus* | 2 | 25.0 | 1.7E+04 | 4.2 | N/A | 0.0E+00 | 0.0 |
| *Ae mediovittatus* | 2 | 32.3 | 3.0E+02 | 2.5 | 36.9 | 1.7E+01 | 1.2 |
| *Ae mediovittatus* | 2 | 29.7 | 1.3E+03 | 3.1 | 40.5 | 2.8E+00 | 0.4 |
| *Ae mediovittatus* | 2 | 26.7 | 6.8E+03 | 3.8 | 43.0 | 7.8E-01 | -0.1 |
| *Ae mediovittatus* | 2 | 31.5 | 4.7E+02 | 2.7 | 33.9 | 7.7E+01 | 1.9 |
| *Ae mediovittatus* | 2 | 32.6 | 2.5E+02 | 2.4 | N/A | 0.0E+00 | 0.0 |
| *Ae mediovittatus* | 2 | 34.2 | 1.0E+02 | 2.0 | N/A | 0.0E+00 | 0.0 |
| *Ae mediovittatus* | 2 | 35.7 | 4.7E+01 | 1.7 | N/A | 0.0E+00 | 0.0 |
| *Ae mediovittatus* | 2 | 29.5 | 1.4E+03 | 3.1 | 36.5 | 2.1E+01 | 1.3 |
| *Ae mediovittatus* | 2 | 32.6 | 2.6E+02 | 2.4 | N/A | 0.0E+00 | 0.0 |
| *Ae mediovittatus* | 2 | 32.5 | 2.8E+02 | 2.4 | 35.3 | 3.9E+01 | 1.6 |

**Table S3**

| **Sample data** | | **Body (infection) data** | | | **Saliva (transmission) data** | | |
| --- | --- | --- | --- | --- | --- | --- | --- |
|  |  | **only positive mosquitoes** | | |  |  |  |
| **mosquito** | **DENV** | **Ct** | **titer** | **log** | **Ct** | **titer** | **log** |
| **species** | **serotype** |  | **log10 pfu/** | **titer** |  | **log10 pfu/** | **titer** |
|  |  |  | **mosquito** |  |  | **mosquito** |  |
| *Ae aegypti* | 3 | 35.6 | 1.8E+00 | 0.3 | 33.6 | 2.5E+00 | 0.4 |
| *Ae aegypti* | 3 | 29.7 | 5.5E+01 | 1.7 | 38.2 | 1.7E-01 | -0.8 |
| *Ae aegypti* | 3 | 33.5 | 5.9E+00 | 0.8 | 36.7 | 3.9E-01 | -0.4 |
| *Ae aegypti* | 3 | 21.0 | 8.9E+03 | 4.0 | 38.9 | 1.1E-01 | -1.0 |
| *Ae aegypti* | 3 | 24.5 | 1.2E+03 | 3.1 | 35.8 | 7.0E-01 | -0.2 |
| *Ae aegypti* | 3 | 27.2 | 2.3E+02 | 2.4 | 35.8 | 7.0E-01 | -0.2 |
| *Ae mediovittatus* | 3 | 35.2 | 1.7E+00 | 0.2 | 38.0 | 1.8E-01 | -0.7 |
| *Ae mediovittatus* | 3 | 34.8 | 2.2E+00 | 0.3 | N/A | 0.0E+00 | 0.0 |
| *Ae mediovittatus* | 3 | 35.4 | 1.6E+00 | 0.2 | 40.7 | 3.8E-02 | -1.4 |
| *Ae mediovittatus* | 3 | 35.2 | 1.7E+00 | 0.2 | N/A | 0.0E+00 | 0.0 |
| *Ae mediovittatus* | 3 | 34.8 | 2.2E+00 | 0.3 | 35.6 | 7.7E-01 | -0.1 |
| *Ae mediovittatus* | 3 | 35.2 | 1.7E+00 | 0.2 | 38.0 | 1.9E-01 | -0.7 |
| *Ae mediovittatus* | 3 | 34.2 | 3.1E+00 | 0.5 | N/A | 0.0E+00 | 0.0 |
| *Ae mediovittatus* | 3 | 34.4 | 2.8E+00 | 0.5 | 38.2 | 1.7E-01 | -0.8 |
| *Ae mediovittatus* | 3 | 21.8 | 4.5E+03 | 3.6 | 34.8 | 1.2E+00 | 0.1 |
| *Ae mediovittatus* | 3 | 26.4 | 3.0E+02 | 2.5 | 20.6 | 5.7E+03 | 3.8 |
| *Ae mediovittatus* | 3 | 25.6 | 4.7E+02 | 2.7 | 41.1 | 3.0E-02 | -1.5 |

**Table S4**

| **Sample data** | | **Body (infection) data** | | | **Saliva (transmission) data** | | |
| --- | --- | --- | --- | --- | --- | --- | --- |
|  |  | **only positive mosquitoes** | | |  |  |  |
| **Mosquito** | **DENV** | **Ct** | **titer** | **Log** | **Ct** | **titer** | **Log** |
| **species** | **serotype** |  | **log10 pfu/** | **titer** |  | **log10 pfu/** | **titer** |
|  |  |  | **mosquito** |  |  | **mosquito** |  |
| *Ae aegypti* | 4 | 31.9 | 6.5E+02 | 2.8 | 39.7 | 1.6E+00 | 0.2 |
| *Ae aegypti* | 4 | 32.7 | 4.4E+02 | 2.6 | 32.0 | 9.7E+01 | 2.0 |
| *Ae aegypti* | 4 | 27.8 | 5.0E+03 | 3.7 | 38.6 | 2.9E+00 | 0.5 |
| *Ae aegypti* | 4 | 28.5 | 3.5E+03 | 3.5 | 31.5 | 1.3E+02 | 2.1 |
| *Ae aegypti* | 4 | 24.0 | 3.3E+04 | 4.5 | 42.0 | 4.5E-01 | -0.4 |
| *Ae aegypti* | 4 | 25.8 | 1.4E+04 | 4.1 | 31.2 | 1.6E+02 | 2.2 |
| *Ae aegypti* | 4 | 22.9 | 5.9E+04 | 4.8 | 41.6 | 5.5E-01 | -0.3 |
| *Ae aegypti* | 4 | 26.4 | 1.0E+04 | 4.0 | 32.8 | 6.5E+01 | 1.8 |
| *Ae aegypti* | 4 | 25.2 | 1.9E+04 | 4.3 | 32.0 | 1.0E+02 | 2.0 |
| *Ae aegypti* | 4 | 24.6 | 2.5E+04 | 4.4 | 40.1 | 1.3E+00 | 0.1 |
| *Ae aegypti* | 4 | 23.8 | 3.8E+04 | 4.6 | N/A | 0.0E+00 | 0.0 |
| *Ae aegypti* | 4 | 30.0 | 1.7E+03 | 3.2 | N/A | 0.0E+00 | 0.0 |
| *Ae aegypti* | 4 | 31.9 | 6.6E+02 | 2.8 | N/A | 0.0E+00 | 0.0 |
| *Ae aegypti* | 4 | 30.4 | 1.4E+03 | 3.2 | 33.4 | 4.6E+01 | 1.7 |
| *Ae aegypti* | 4 | 33.2 | 3.5E+02 | 2.5 | 35.5 | 1.5E+01 | 1.2 |
| *Ae aegypti* | 4 | 32.5 | 4.9E+02 | 2.7 | 33.0 | 5.9E+01 | 1.8 |
| *Ae aegypti* | 4 | 30.6 | 1.3E+03 | 3.1 | N/A | 0.0E+00 | 0.0 |
| *Ae aegypti* | 4 | 29.0 | 2.9E+03 | 3.5 | 29.3 | 4.3E+02 | 2.6 |
| *Ae aegypti* | 4 | 30.3 | 1.5E+03 | 3.2 | 30.9 | 1.8E+02 | 2.2 |

**Table S5**

| **Sample data** | | **Body (infection) data** | | | **Saliva (transmission) data** | | |
| --- | --- | --- | --- | --- | --- | --- | --- |
|  |  | **only positive mosquitoes** | | |  |  |  |
| **Mosquito** | **DENV** | **Ct** | **titer** | **Log** | **Ct** | **titer** | **Log** |
| **species** | **serotype** |  | **log10 pfu/** | **titer** |  | **log10 pfu/** | **titer** |
|  |  |  | **mosquito** |  |  | **mosquito** |  |
| *Ae aegypti* | 4 | 32.1 | 6.0E+02 | 2.8 | 33.9 | 3.5E+01 | 1.5 |
| *Ae aegypti* | 4 | 26.8 | 8.2E+03 | 3.9 | 31.7 | 1.1E+02 | 2.1 |
| *Ae aegypti* | 4 | 36.4 | 7.1E+01 | 1.8 | 30.9 | 1.8E+02 | 2.2 |
| *Ae aegypti* | 4 | 33.7 | 2.7E+02 | 2.4 | 34.6 | 2.5E+01 | 1.4 |
| *Ae aegypti* | 4 | 28.9 | 3.0E+03 | 3.5 | 34.9 | 2.1E+01 | 1.3 |
| *Ae aegypti* | 4 | 33.3 | 3.3E+02 | 2.5 | 34.4 | 2.8E+01 | 1.4 |
| *Ae aegypti* | 4 | 31.1 | 1.0E+03 | 3.0 | 29.6 | 3.6E+02 | 2.6 |
| *Ae aegypti* | 4 | 32.4 | 5.1E+02 | 2.7 | 34.4 | 2.7E+01 | 1.4 |
| *Ae aegypti* | 4 | 31.5 | 8.3E+02 | 2.9 | 34.1 | 3.2E+01 | 1.5 |
| *Ae aegypti* | 4 | 28.1 | 4.5E+03 | 3.7 | N/A | 0.0E+00 | 0.0 |
| *Ae aegypti* | 4 | 32.8 | 4.3E+02 | 2.6 | N/A | 0.0E+00 | 0.0 |
| *Ae aegypti* | 4 | 31.9 | 6.8E+02 | 2.8 | N/A | 0.0E+00 | 0.0 |
| *Ae aegypti* | 4 | 33.6 | 2.9E+02 | 2.5 | 30.2 | 2.6E+02 | 2.4 |
| *Ae aegypti* | 4 | 31.1 | 9.8E+02 | 3.0 | 33.3 | 4.9E+01 | 1.7 |
| *Ae aegypti* | 4 | 28.9 | 3.0E+03 | 3.5 | 31.1 | 1.7E+02 | 2.2 |
| *Ae aegypti* | 4 | 30.5 | 1.3E+03 | 3.1 | 34.5 | 2.6E+01 | 1.4 |
| *Ae aegypti* | 4 | 39.3 | 1.7E+01 | 1.2 | 34.8 | 2.2E+01 | 1.4 |
| *Ae aegypti* | 4 | 28.9 | 3.0E+03 | 3.5 | 31.5 | 1.3E+02 | 2.1 |
| *Ae mediovittatus* | 4 | 28.8 | 1.3E+03 | 3.1 | 28.0 | 8.5E+02 | 2.9 |
